# Prognostic, clinicopathological, and immune correlation of NLRP3 promoter methylation in kidney renal clear cell carcinoma

**DOI:** 10.1002/ctm2.528

**Published:** 2021-10-21

**Authors:** Qian Long, Liru He, Jin Peng, Qi Meng, Changlin Zhang, Miao Chen, Xiaonan Wang, Wancui Zhu, Fufu Zheng, Pei Dong, Wuguo Deng

**Affiliations:** ^1^ State Key Laboratory of Oncology in South China, Collaborative Innovation Center of Cancer Medicine Sun Yat‐sen University Cancer Center Guangzhou China; ^2^ Department of Gynecology The Seventh Affiliated Hospital of Sun Yat‐sen University Shenzhen China; ^3^ The First Affiliated Hospital of Sun Yat‐sen University Guangzhou China

**Abbreviations**
CPTAC: The National Cancer Institute's Clinical Proteomic Tumor Analysis Consortium
GEO: Gene Expression Omnibus
IHC: immunohistochemistry
KIRC: kidney renal clear cell carcinoma
NLRP3: Nod‐like receptor protein 3
ssGSEA: single‐sample gene‐set enrichment analysis
SYSUCC: Sun Yat‐sen University Cancer Center
TCGA: The Cancer Genome Atlas

Dear Editor,

NLRP3 is a cytosolic protein, which plays a crucial role in inflammatory response.^1^ Abnormal elevation of NLRP3 expression has been observed in a variety of cancers and associated with tumor progression.^2^ However, systematic analysis of NLRP3 promoter methylation in cancers remains elusive as yet. Hence, we analyzed the NLRP3 promoter methylation between normal and tumor tissues in all tumor types from TCGA database, and we found that NLRP3 promoter was hypomethylated in tumor tissues in the majority of tumor types (Figure S1). Subsequently, we analyzed the prognostic value of NLPR3 promoter by univariate Cox regression and found that NLRP3 promoter methylation had the best predictive efficiency of overall survival in kidney renal clear cell carcinoma (KIRC) patients (Table S1). Therefore, we focused on investigating the role of NLRP3 promoter methylation in KIRC.

KIRC is considered to be an immunotherapy‐responsive tumor,^3^ and the management of KIRC has been transformed by the development of immune‐checkpoint inhibitors.^4^ Here, we aim to investigate the immune correlation and clinical significance of NLRP3 promoter methylation in KIRC. To investigate NLRP3 promoter methylation status in tumor and normal adjacent tissues in KIRC, we conducted the differential analysis of all CpG sites located in NLRP3 in TCGA cohort. We found that all CpG sites in NLRP3 promoter were significantly hypomethylated in KIRC tumor tissues (Figure 1A and Figure S2A). Analysis of two independent GEO datasets further validated hypomethylation of NLRP3 promoter in tumor (Figure S2B, C). Gene promoter methylation plays an important role in gene transcription and expression in both physiological and pathological conditions.^5^ Further investigation of the relationship between NLPR3 expression and its promoter methylation found that NLRP3 expression was significantly elevated in tumor tissues and NLRP3 promoter methylation was significantly negatively correlated with NLPR3 expression (Figure S3). Subsequently, we analyzed the prognostic values of NLRP3 promoter methylation by Kaplan–Meier survival analysis. We found that all hypomethylation of all CpG sites (except cg14413862) was associated with poor survival (Figure 1B–I). Further analysis of the correlation of NLRP3 promoter methylation with clinicopathological characteristics found that NLRP3 promoter was hypomethylated in advanced AJCC stage, TNM stage, and pathological grade (Figure S4).

**FIGURE 1:**
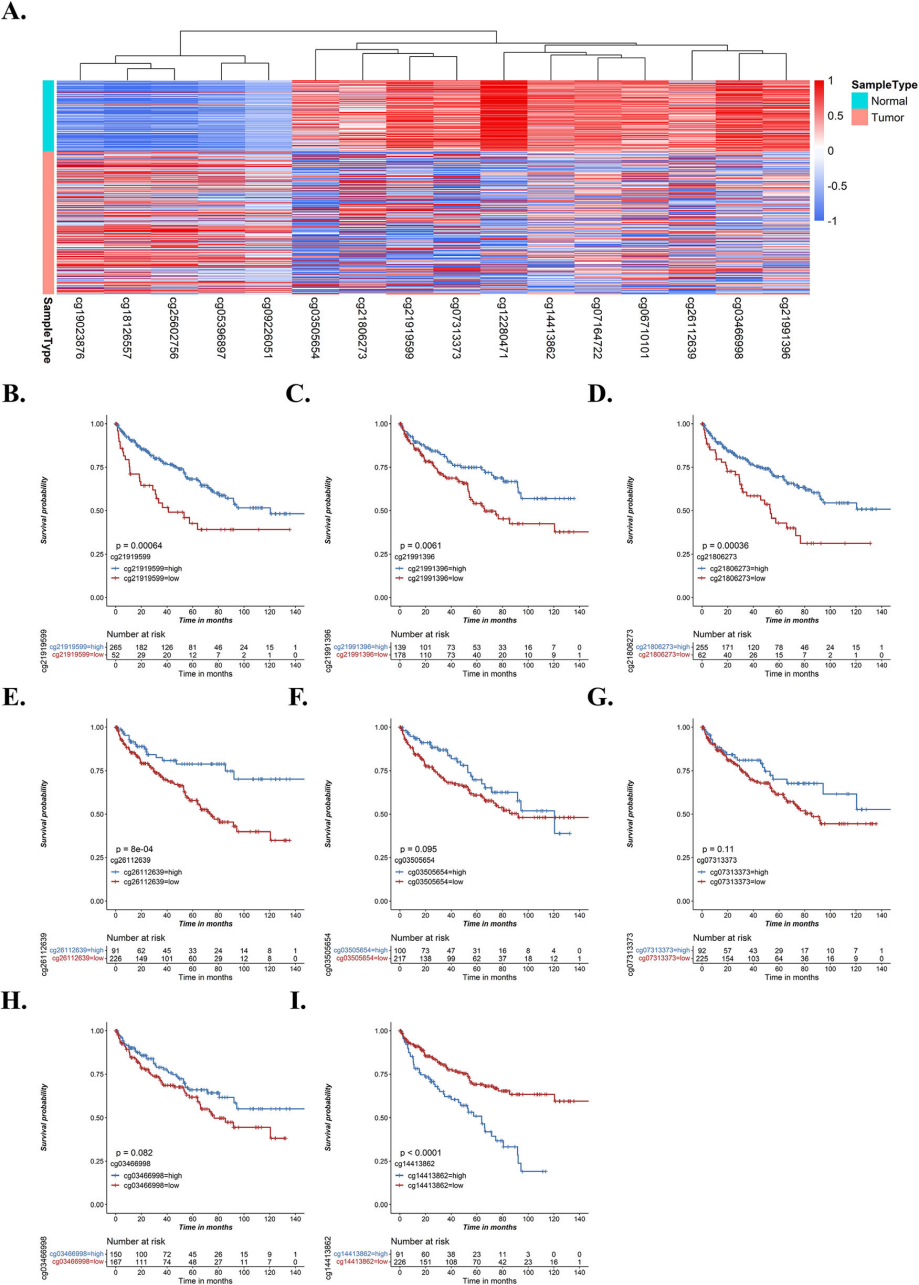
NLRP3 promoter hypomethylation is associated with poor survival in KIRC patients. (A) The heatmap of differentially methylated CpG sites located in NLPR3. (B–I) Kaplan–Meier survival analysis of eight NLRP3 promoter CpG sites (cg21919599, cg21991396, cg26112639, cg21806273, cg03505654, cg07313373, cg03466998, and cg14413862) for overall survival in TCGA cohort according to the optimal cutoff value, respectively

Epigenetic biomarkers, especially DNA methylation, open new avenues for research on clinical biomarkers for immune checkpoint blockade therapy.^6^ Considering the vital role of NLRP3 in immune regulation,^7^ we speculated that NLRP3 was associated with immune cell infiltration of tumor microenvironment. We analyzed the association between NLRP3 expression and promoter methylation with 23 types of immune cells in TCGA KIRC cohort, and we found that NLRP3 expression was significantly positively correlated with infiltration of all types of immune cells in tumor tissues, while NLRP3 promoter methylation (especially cg21919599, cg21991396, cg26112639, and cg21806273) was negatively associated with immune cell infiltration (Figure 2A). This was further validated by our analysis of CPTAC cohort (Figure S5A). To further analyze the role of NLRP3 promoter in tumor microenvironment, we calculated the enrichment scores of three immune suppressive pathways (coinhibition APC, coinhibition T cell, and immune checkpoint) using data from TCGA cohort. We then analyzed the enrichment scores in high and low groups of NLRP3 expression and promoter methylation, which were divided by the median of each, respectively. We found that samples with high NLRP3 expression had significantly elevated enrichment scores (Figure 2B). The enrichment scores of the three pathways were significantly higher in the hypomethylation group (especially in cg21919599, cg21991396, cg26112639, and cg21806273) (Figure 2C–F), whereas no significant difference was found between high and low group of other CpG sites (Figure 2G–I). Our analysis using data from CPTAC cohort also validated our results above (Figure S5B–F). These results demonstrated that NLRP3 expression and promoter methylation were associated with both immune cell infiltration and immune suppressive pathways in KIRC.

**FIGURE 2:**
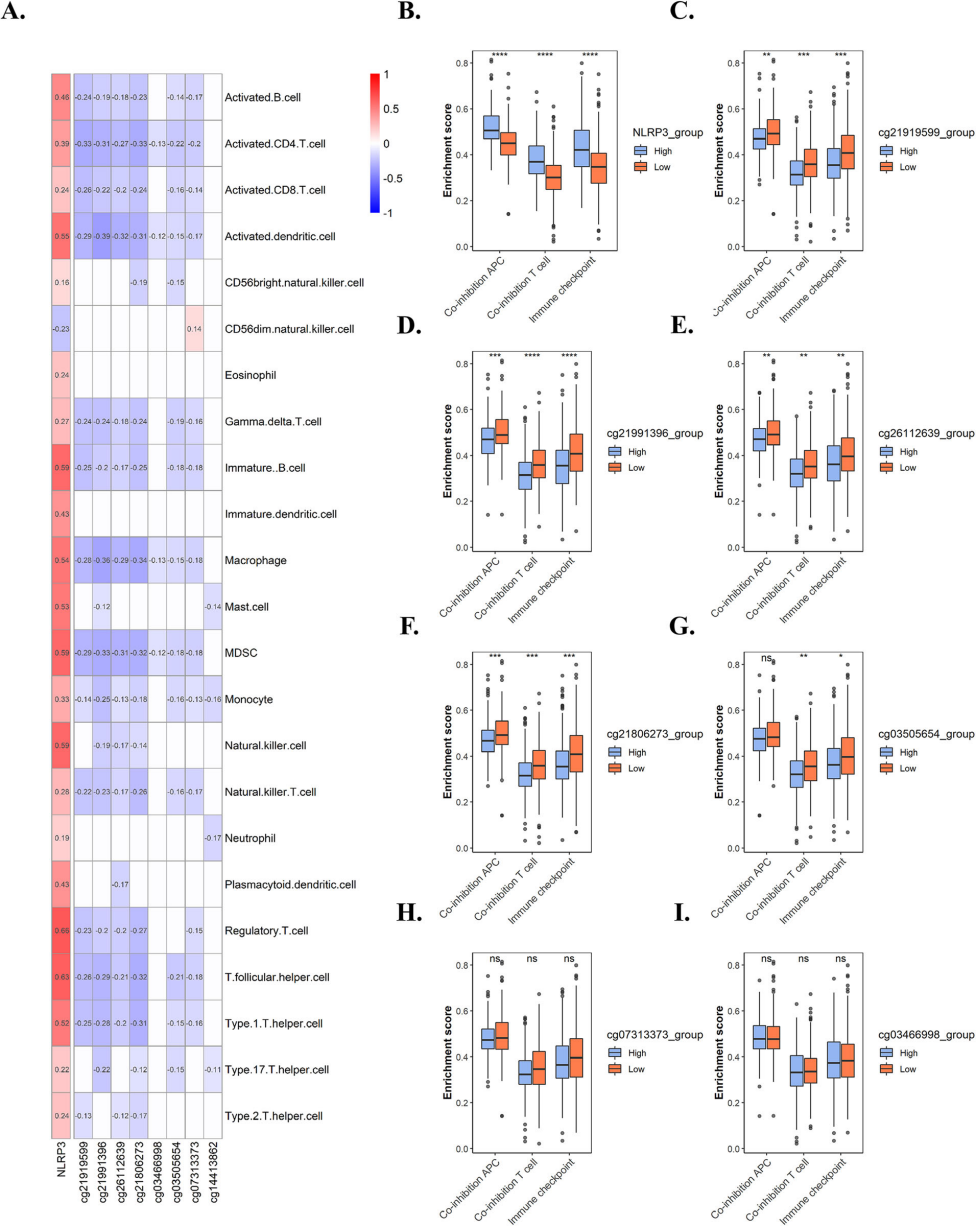
NLRP3 expression and promoter methylation are correlated with immune cell infiltration in KIRC. (A) The correlation heatmap of NLRP3 expression and eight differentially methylated CpG sites with 23 types of immune cells in TCGA KIRC cohort, only statistically significant (*p* < 0.05) are shown in color and correlation coefficients. (B–D) Relative enrichment scores of three immune inhibitory pathways (coinhibition APC, coinhibition T cell, and immune checkpoint) in high and low group according to median of NLRP3 expression and methylation of cg21919599, cg21991396, cg26112639, cg21806273, cg03505654, cg07313373, and cg03466998, respectively

Effective antitumor immune responses were mediated by a variety of cytokines and membrane proteins.^8^ Therefore, we analyzed the association of NLRP3 expression and promoter methylation with 74 types of proteins closely related to regulation of antitumor immune responses using data from TCGA cohort. We found that NLRP3 expression was positively correlated with most of these proteins, especially immune checkpoint molecules, while NLRP3 promoter methylation was negatively associated with immune checkpoint molecules (Figure 3A). Subsequently, we elaborately analyzed the association of NLRP3 expression and methylation of cg21919599 and cg21806273 with the most important immune checkpoint molecules (LAG3, PD1, CD80, TIGIT, PDL2, and CTLA4), and we found that NLRP3 expression was positively correlated with these checkpoint molecules, whereas methylation of cg21919599 and cg21806273 was negatively correlated (Figure 3B–D). Moreover, our analysis using data from CPTAC also validated our results (Figure S6A–C).

**FIGURE 3:**
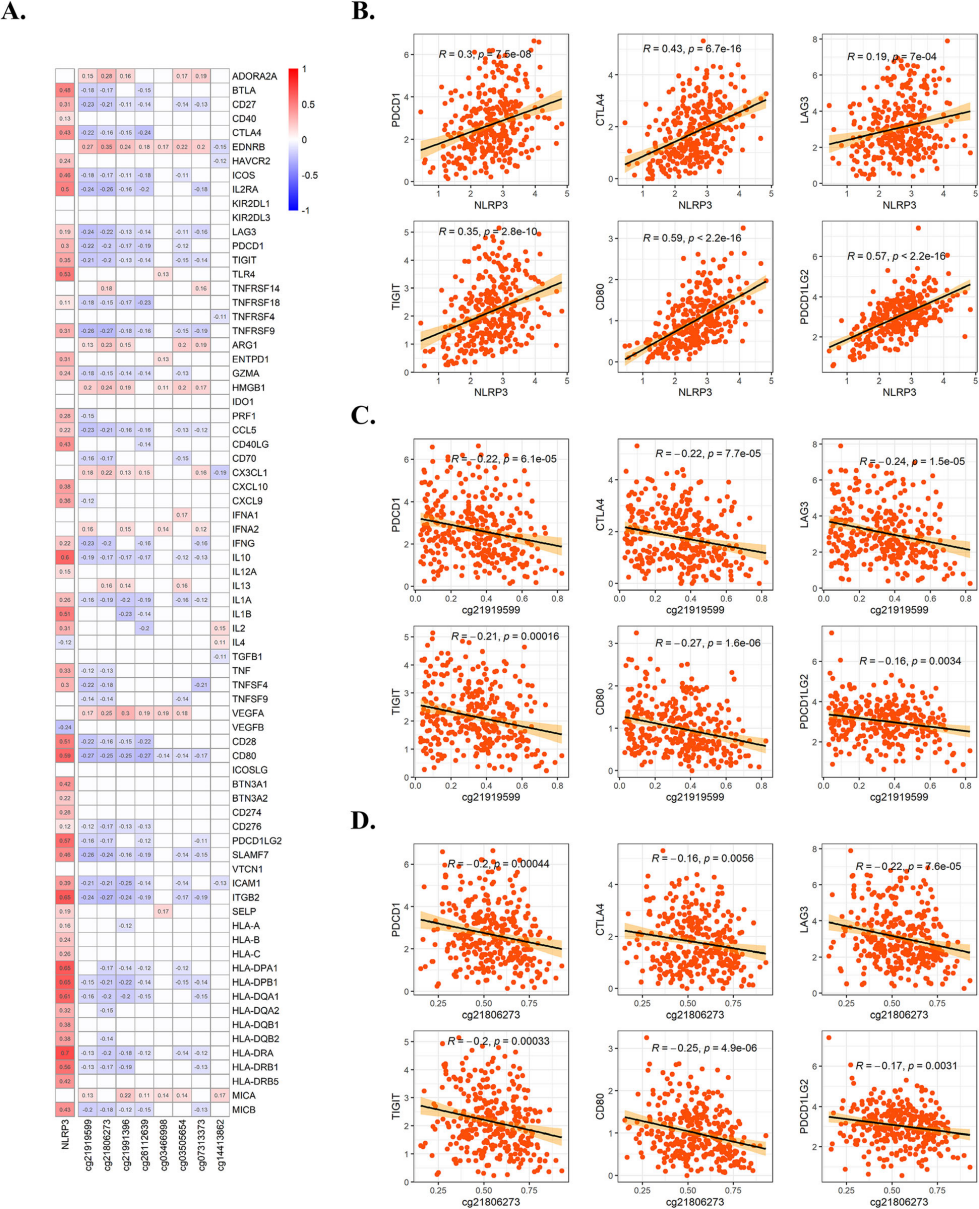
NLRP3 expression and promoter methylation correlate with the expression of immune checkpoint molecules. (A) The correlation heatmap of NLRP3 expression and eight differentially methylated CpG sites with 74 types of proteins closely related to regulation of antitumor immune responses in TCGA KIRC cohort, only statistically significant (*p* < 0.05) are shown in color and correlation coefficients. (B–D) The correlation of NLRP3 expression and cg21919599 or cg21806273 methylation with immune checkpoint molecules PD1, CTLA4, LAG3, TIGIT, CD80, and PDL2, respectively

Considering that cg21919599 was among the most significant prognostic factors and its significant association with TNM stage, pathological grade, and infiltration of immune cell, we selected CpG site cg21919599 for our verification in SYSUCC validation cohort. We also found significant positive correlation between cg21919599 and three promising CpG sites (cg21806273, cg21991396, and cg26112639) (Figure S7), indicating that cg21919599 was representative. First, we examined the methylation of cg21919599 by pyrosequencing. The results showed that methylation of cg21919599 was significantly decreased in tumors compared to normal adjacent tissues (Figure 4A) and cg21919599 hypomethylation was associated with poor overall survival (Figure 4B). CD4 and CD8 T cells are the final executors of antitumor immune responses, which are the most important immune cells in antitumor immunity.^9^ Hence, we examined the expression of CD4, CD8, and NLRP3 in tumor tissues by immunohistochemistry (IHC). We found that cg21919599 methylation was negatively correlated with CD4, CD8, and NLRP3 expression (Figure 4C, D), which was consistent with our previous results from TCGA and CPTAC cohorts. These results further validated that NLRP3 promoter methylation was associated with overall survival and infiltration of immune cells in KIRC.

**FIGURE 4:**
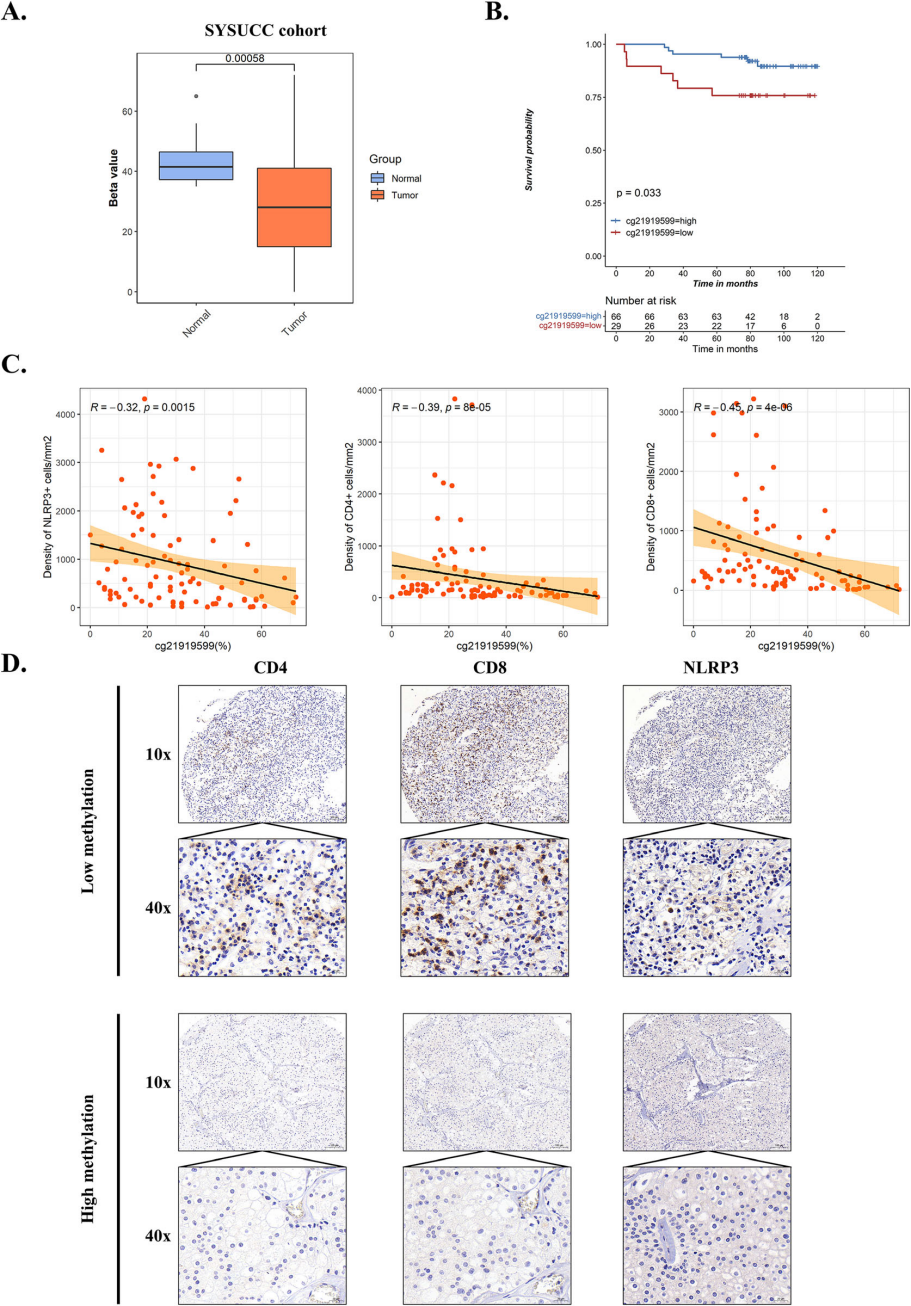
NLRP3 promoter methylation is associated with overall survival and tumor infiltration of CD4/CD8 T cells in SYSUCC validation cohort. (A) Relative cg21919599 methylation between tumor and normal adjacent tissues of SYSYCC cohort. (B) Kaplan–Meier survival analysis of cg21919599 methylation in SYSUCC cohort. (C) The correlation of cg21919599 methylation with CD4, CD8, and NLRP3 expression in SYSUCC cohort, respectively. (D) The representative IHC pictures of CD4, CD8, and NLRP3 in high and low cg21919599 methylation group of SYSUCC cohort (high group: 66, low group: 29)

In conclusion, our data suggested that NLRP3 mRNA expression was probably regulated by its promoter methylation, which is associated with clinicopathological characteristics, overall survival, and immune cell infiltration in KIRC. Our study provides a solid ground for further testing NLRP3 promoter methylation as a predictive biomarker for response to immune checkpoint inhibitors in KIRC.

## COMPETING INTERESTS

The authors have declared that no competing interests exist.

## ETHICS APPROVAL AND CONSENT TO PARTICIPATE

The clinical specimens were obtained from Tumor Bio‐bank of Sun Yat‐sen University Cancer Center. The study protocol for the SYSUCC cohort was approved by the Institutional Research Ethics Committee of Sun Yat‐sen University Cancer Center (B2020‐310‐01).

## AUTHOR CONTRIBUTIONS

QL, LH, PD, and WD conceived and designed the project. QL, CZ, QM, MC, XW, WZ, JP, and DS performed the experiments and analyzed and interpreted the data. QL, LH, PD, and WD wrote and revised the manuscript. All authors read and approved the final manuscript.

## DATA AVAILABILITY STATEMENT

All data generated or analyzed during this study are included either in this article or in the additional files.

## Supporting information

Figure S1. The mean methylation of NLRP3 promoter in tumor and normal tissues in pan cancer using TCGA data.
Figure S2. NLRP3 promoter is hypomethylated in KIRC tumor tissues. (A) The methylation level of eight NLRP3 promoter CpG sites (cg21919599, cg21991396, cg26112639, cg21806273, cg03505654, cg07313373, cg03466998, and cg14413862) between tumor and normal adjacent tissues in TCGA cohort. (B, C) The methylation level of eight NLRP3 promoter CpG sites (cg21919599, cg21991396, cg26112639, cg21806273, cg03505654, cg07313373, cg03466998, and cg14413862) between tumor and normal adjacent tissues in GSE70303 and GSE105260, respectively.
Figure S3. NLRP3 promoter methylation is negatively correlated with NLRP3 expression in KIRC. (A) The NLRP3 mRNA expression between tumor and normal adjacent tissues (normal: 72, tumor: 539). (B–I) The correlation of methylation of eight NLRP3 promoter CpG sites (cg21919599, cg21991396, cg26112639, cg21806273, cg03505654, cg07313373, cg03466998, and cg14413862) with NLRP3 expression, respectively.
Figure S4. NLRP3 promoter methylation correlates with clinicopathological characteristics in KIRC. (A–E) Relative methylation level of eight NLRP3 promoter CpG sites (cg21919599, cg21991396, cg26112639, cg21806273, cg03505654, cg07313373, cg03466998, and cg14413862) in different TNM stage and pathological grade of patients from TCGA cohort.
Figure S5. NLRP3 expression and promoter methylation correlate with immune cell infiltration in CPTAC KIRC cohort. (A) The correlation heatmap of NLRP3 expression and eight differentially methylated CpG sites with 23 types of immune cells in CPTAC KIRC cohort, only statistically significant (*p* < 0.05) are shown in color and correlation coefficients. (B–F) The correlation of enrichment scores of three immune inhibitory pathways (coinhibition APC, coinhibition T cell, and immune checkpoint) with NLRP3 expression and methylation of cg21919599, cg21991396, cg21806273, and cg26112639, respectively.
Figure S6. NLRP3 expression and promoter methylation correlate with the expression of immune checkpoint molecules in CPTAC KIRC cohort. (A–C) The correlation of NLRP3 expression and cg21919599 or cg21806273 methylation with immune checkpoint molecules PD1, CTLA4, LAG3, TIGIT, CD80, and PDL2, respectively.
Figure S7. The correlation of cg21919599 with other promising CpG sites in TCGA cohort. (A–C) The correlation of cg21919599 methylation with methylation of cg21806273, cg21991396, and cg26112639, respectively.

FigureS1

FigureS2

FigureS3

FigureS4

FigureS5

FigureS6

FigureS7

Supporting Information

TablesS1‐S9

## References

[1] Paik S , Kim JK , Silwal P , Sasakawa C , Jo EK . An update on the regulatory mechanisms of NLRP3 inflammasome activation. Cell Mol Immunol. 2021;18(5):1141‐1160. 33850310 10.1038/s41423-021-00670-3 PMC8093260

[2] Sharma BR , Kanneganti TD . NLRP3 inflammasome in cancer and metabolic diseases. Nat Immunol. 2021;22:550‐559. 33707781 10.1038/s41590-021-00886-5 PMC8132572

[3] Diaz‐Montero CM , Rini BI , Finke JH . The immunology of renal cell carcinoma. Nat Rev Nephrol. 2020;16(12):721‐735. 32733094 10.1038/s41581-020-0316-3

[4] Braun DA , Bakouny Z , Hirsch L , et al. Beyond conventional immune‐checkpoint inhibition — novel immunotherapies for renal cell carcinoma. Nat Rev Clin Oncol. 2021;18(4):199‐214. 33437048 10.1038/s41571-020-00455-z PMC8317018

[5] Luo C , Hajkova P , Ecker JR . Dynamic DNA methylation: in the right place at the right time. Science. 2018;361(6409):1336‐1340. 30262495 10.1126/science.aat6806 PMC6197482

[6] Xiao Q , Nobre A , Pineiro P , et al. Genetic and epigenetic biomarkers of immune checkpoint blockade response. J Clin Med. 2020;9(1):286. 10.3390/jcm9010286 PMC7019273 31968651

[7] Deets KA , Vance RE . Inflammasomes and adaptive immune responses. Nat Immunol. 2021;22(4):412‐422. 33603227 10.1038/s41590-021-00869-6

[8] Ozga AJ , Chow MT , Luster AD . Chemokines and the immune response to cancer. Immunity. 2021;54:859‐874. 33838745 10.1016/j.immuni.2021.01.012 PMC8434759

[9] Lei X , Lei Y , Li JK , et al. Immune cells within the tumor microenvironment: biological functions and roles in cancer immunotherapy. Cancer Lett. 2020;470:126‐133. 31730903 10.1016/j.canlet.2019.11.009

