# Supplementary material for: Prognostic, clinicopathological, and immune correlation of NLRP3 promoter methylation in kidney renal clear cell carcinoma
Source: Clin Transl Med. 2021 Oct 21;11(10):e528. doi: 10.1002/ctm2.528 (PMC8530444; doi:10.1002/ctm2.528)
Supplement: Supplementary file 1 — Figure S1. The mean methylation of NLRP3 promoter in tumor and normal tissues in pan cancer using TCGA data. Figure S2. NLRP3 promoter is hypomethylated in KIRC tumor tissues. (A) The methylation level of eight NLRP3 promoter CpG sites (cg21919599, cg21991396, cg26112639, cg21806273, cg03505654, cg07313373, cg03466998, and cg14413862) between tumor and normal adjacent tissues in TCGA cohort. (B, C) The methylation level of eight NLRP3 promoter CpG sites (cg21919599, cg21991396, cg26112639, cg21806273, cg03505654, cg07313373, cg03466998, and cg14413862) between tumor and normal adjacent tissues in GSE70303 and GSE105260, respectively. Figure S3. NLRP3 promoter methylation is negatively correlated with NLRP3 expression in KIRC. (A) The NLRP3 mRNA expression between tumor and normal adjacent tissues (normal: 72, tumor: 539). (B–I) The correlation of methylation of eight NLRP3 promoter CpG sites (cg21919599, cg21991396, cg26112639, cg21806273, cg03505654, cg07313373, cg03466998, and cg14413862) with NLRP3 expression, respectively. Figure S4. NLRP3 promoter methylation correlates with clinicopathological characteristics in KIRC. (A–E) Relative methylation level of eight NLRP3 promoter CpG sites (cg21919599, cg21991396, cg26112639, cg21806273, cg03505654, cg07313373, cg03466998, and cg14413862) in different TNM stage and pathological grade of patients from TCGA cohort. Figure S5. NLRP3 expression and promoter methylation correlate with immune cell infiltration in CPTAC KIRC cohort. (A) The correlation heatmap of NLRP3 expression and eight differentially methylated CpG sites with 23 types of immune cells in CPTAC KIRC cohort, only statistically significant (p < 0.05) are shown in color and correlation coefficients. (B–F) The correlation of enrichment scores of three immune inhibitory pathways (coinhibition APC, coinhibition T cell, and immune checkpoint) with NLRP3 expression and methylation of cg21919599, cg21991396, cg21806273, and cg26112639, respectively. Figure S6. NL [file CTM2-11-e528-s004.docx]

**Figure S1. The mean methylation of NLRP3 promoter in tumor and normal tissues in pan cancer using TCGA data.**

F**igure S2. NLRP3 promoter is hypomethylated in KIRC tumor tissues.** A. The methylation level of eight NLRP3 promoter CpG sites (cg21919599, cg21991396, cg26112639, cg21806273, cg03505654, cg07313373, cg03466998 and cg14413862) between tumor and normal adjacent tissues in TCGA cohort; B, C. The methylation level of eight NLRP3 promoter CpG sites (cg21919599, cg21991396, cg26112639, cg21806273, cg03505654, cg07313373, cg03466998 and cg14413862) between tumor and normal adjacent tissues in GSE70303 and GSE105260, respectively.

**Figure S3. NLRP3 promoter methylation is negatively correlated with NLRP3 expression in KIRC.** A. The NLRP3 mRNA expression between tumor and normal adjacent tissues (normal: 72, tumor: 539); B-I. The correlation of methylation of eight NLRP3 promoter CpG sites (cg21919599, cg21991396, cg26112639, cg21806273, cg03505654, cg07313373, cg03466998 and cg14413862) with NLRP3 expression, respectively.

**Figure S4. NLRP3 promoter methylation correlates with clinicopathological characteristics in KIRC.** A-E. Relative methylation level of eight NLRP3 promoter CpG sites (cg21919599, cg21991396, cg26112639, cg21806273, cg03505654, cg07313373, cg03466998 and cg14413862) in different TNM stage and pathological grade of patients from TCGA cohort.

**Figure S5. NLRP3 expression and promoter methylation correlate with immune cell infiltration in CPTAC KIRC cohort.** A. The correlation heatmap of NLRP3 expression and eight differentially methylated CpG sites with 23 types of immune cells in CPTAC KIRC cohort, only statistically significant (P < 0.05) are shown in color and correlation coefficients. B-F. The correlation of enrichment scores of three immune inhibitory pathways (Co-inhibition APC, Co-inhibition T cell and Immune checkpoint) with NLRP3 expression and methylation of cg21919599, cg21991396, cg21806273, cg26112639, respectively.

**Figure S6. NLRP3 expression and promoter methylation correlate with the expression of immune checkpoint molecules in CPTAC KIRC cohort.** A-C. The correlation of NLRP3 expression and cg21919599 or cg21806273 methylation with immune checkpoint molecules PD1, CTLA4, LAG3, TIGIT, CD80 and PDL2, respectively.

**Figure S7. The correlation of cg21919599 with other promising CpG sites in TCGA cohort.** A-C. The correlation of cg21919599 methylation with methylation of cg21806273, cg21991396 and cg26112639, respectively.
