# Supplementary figures and images for: Prognostic, clinicopathological, and immune correlation of NLRP3 promoter methylation in kidney renal clear cell carcinoma
Source: Clin Transl Med. 2021 Oct 21;11(10):e528. doi: 10.1002/ctm2.528 (PMC8530444; doi:10.1002/ctm2.528)

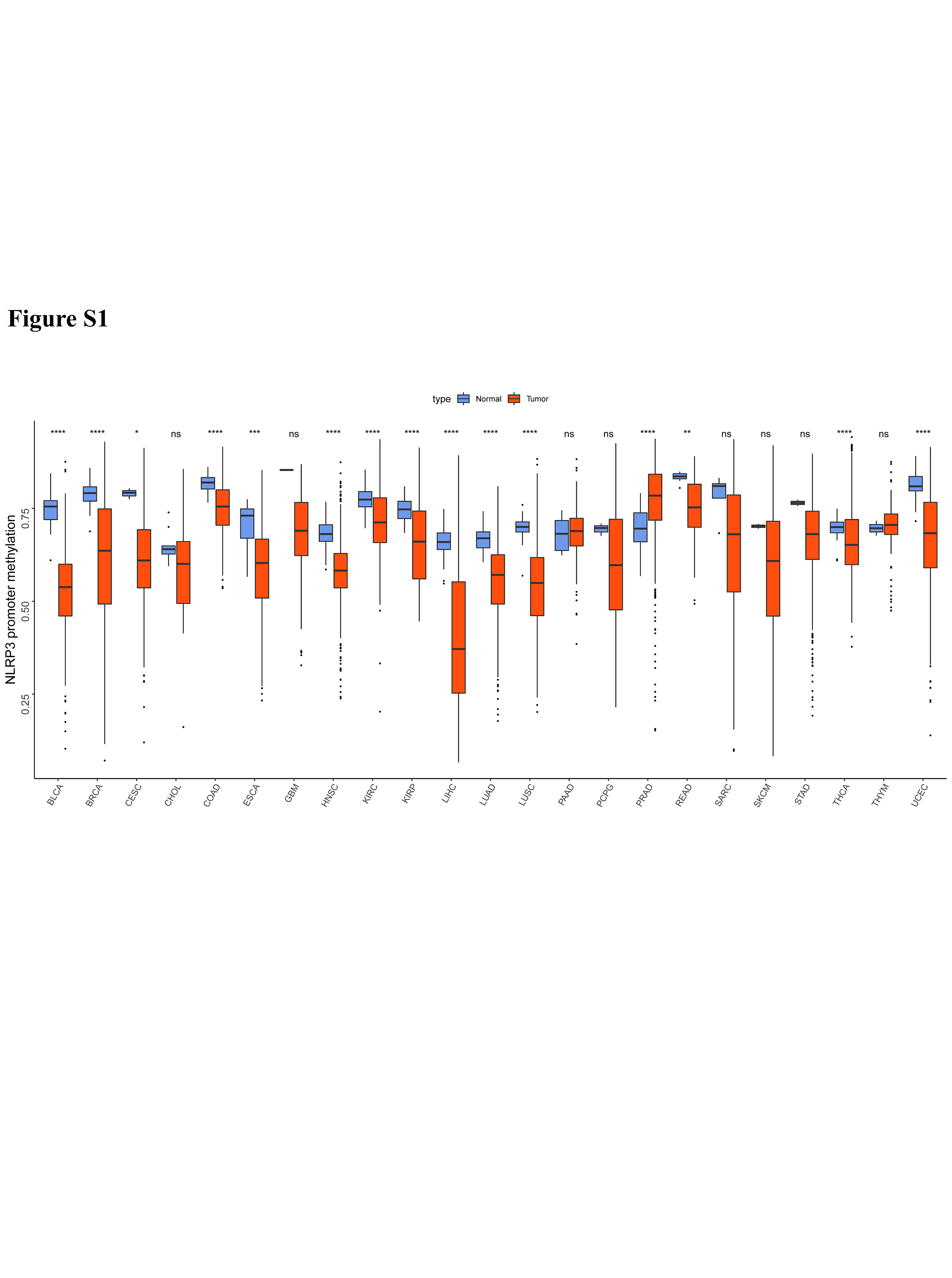

Supplement: Supplementary file 2 — FigureS1 [file CTM2-11-e528-s003.tif]

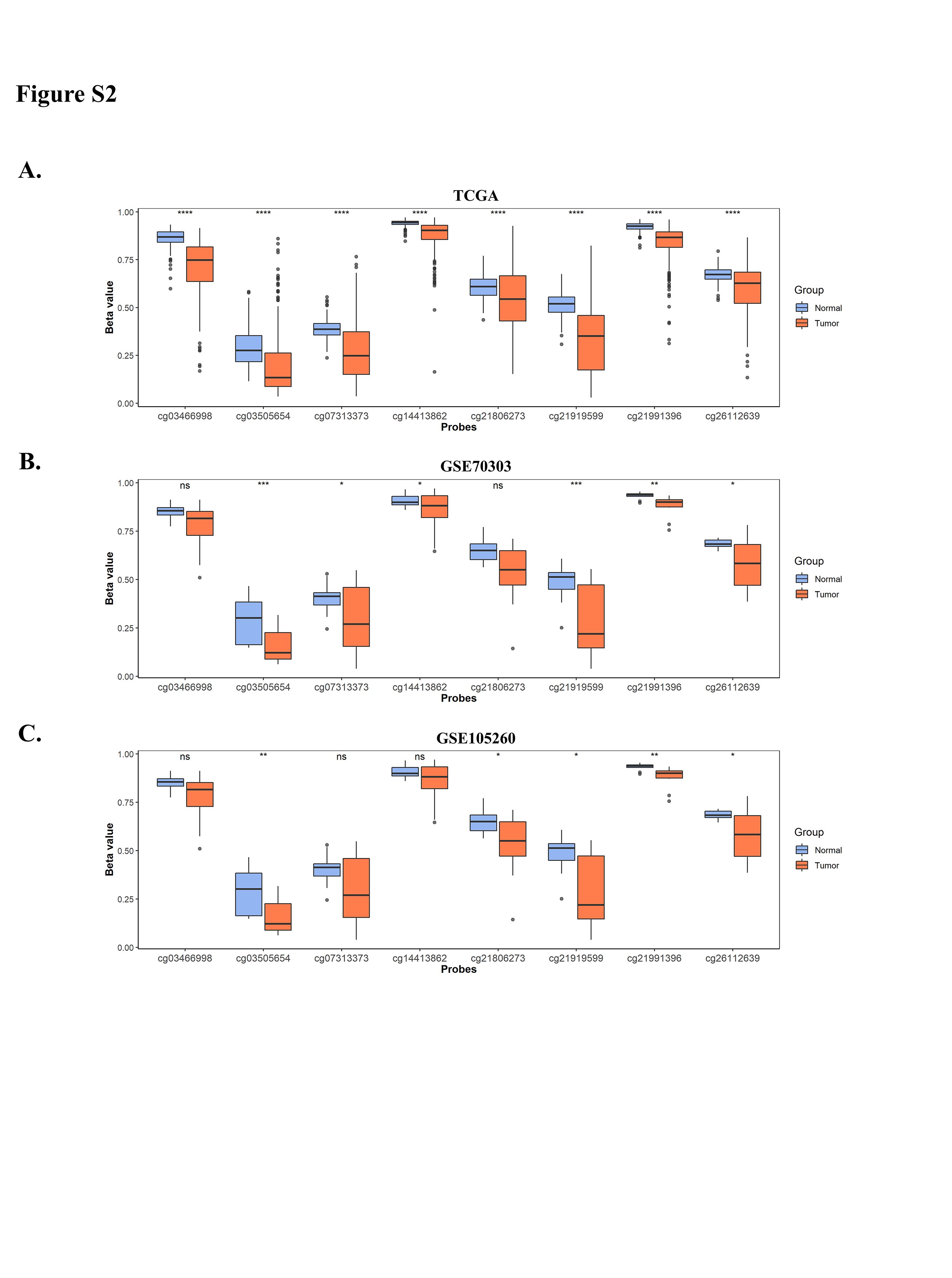

Supplement: Supplementary file 3 — FigureS2 [file CTM2-11-e528-s007.tif]

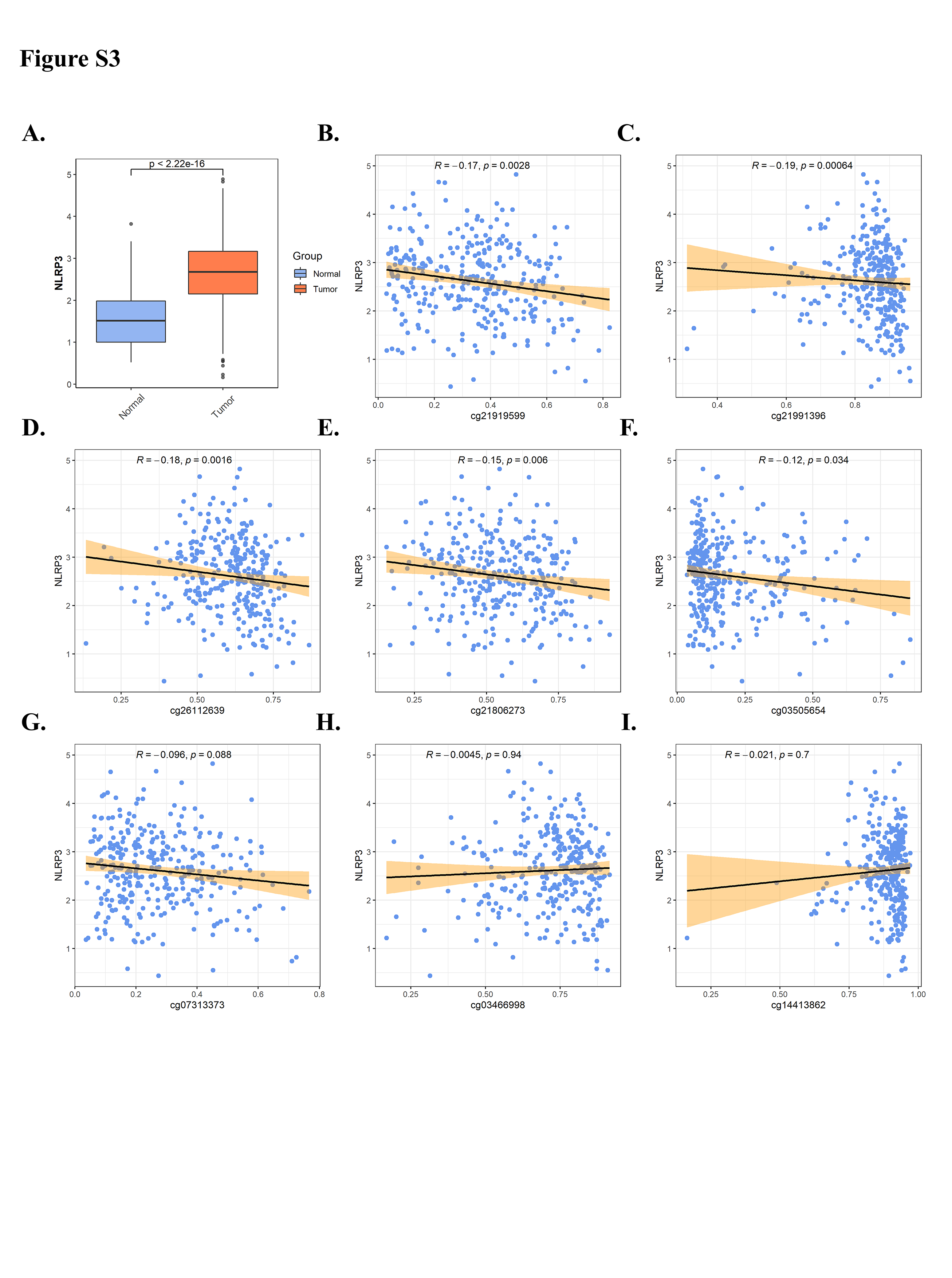

Supplement: Supplementary file 4 — FigureS3 [file CTM2-11-e528-s009.tif]

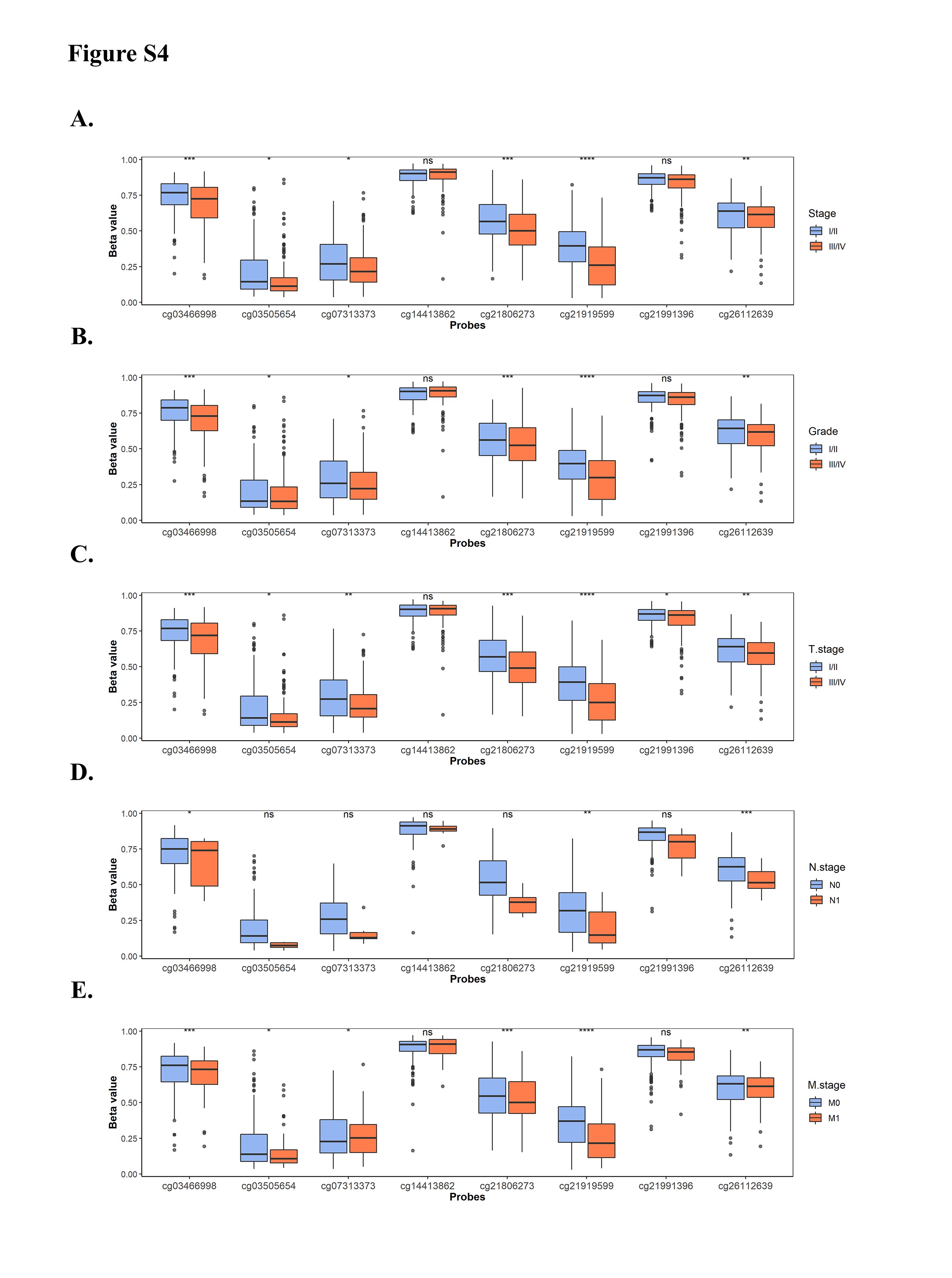

Supplement: Supplementary file 5 — FigureS4 [file CTM2-11-e528-s010.tif]

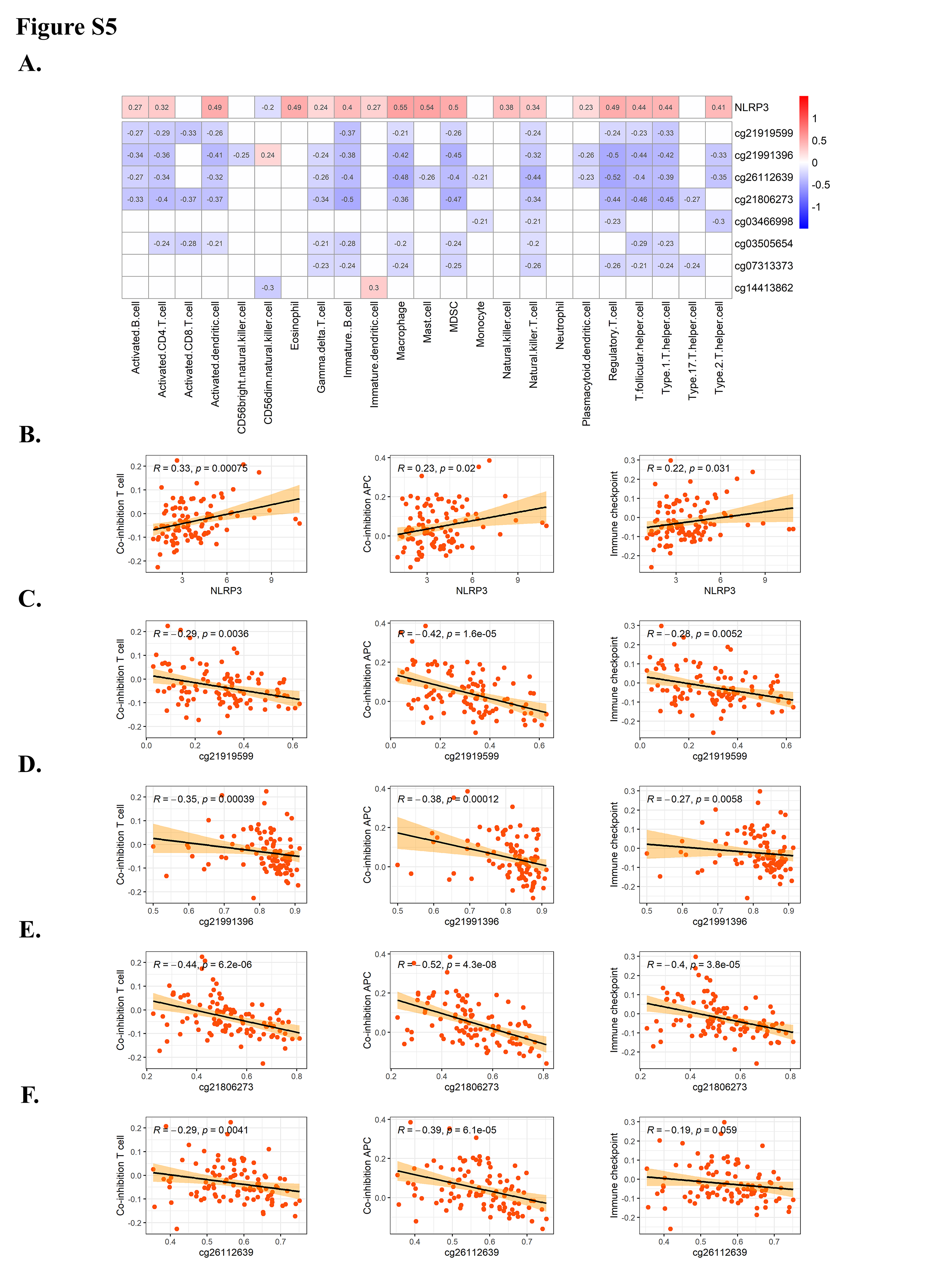

Supplement: Supplementary file 6 — FigureS5 [file CTM2-11-e528-s008.tif]

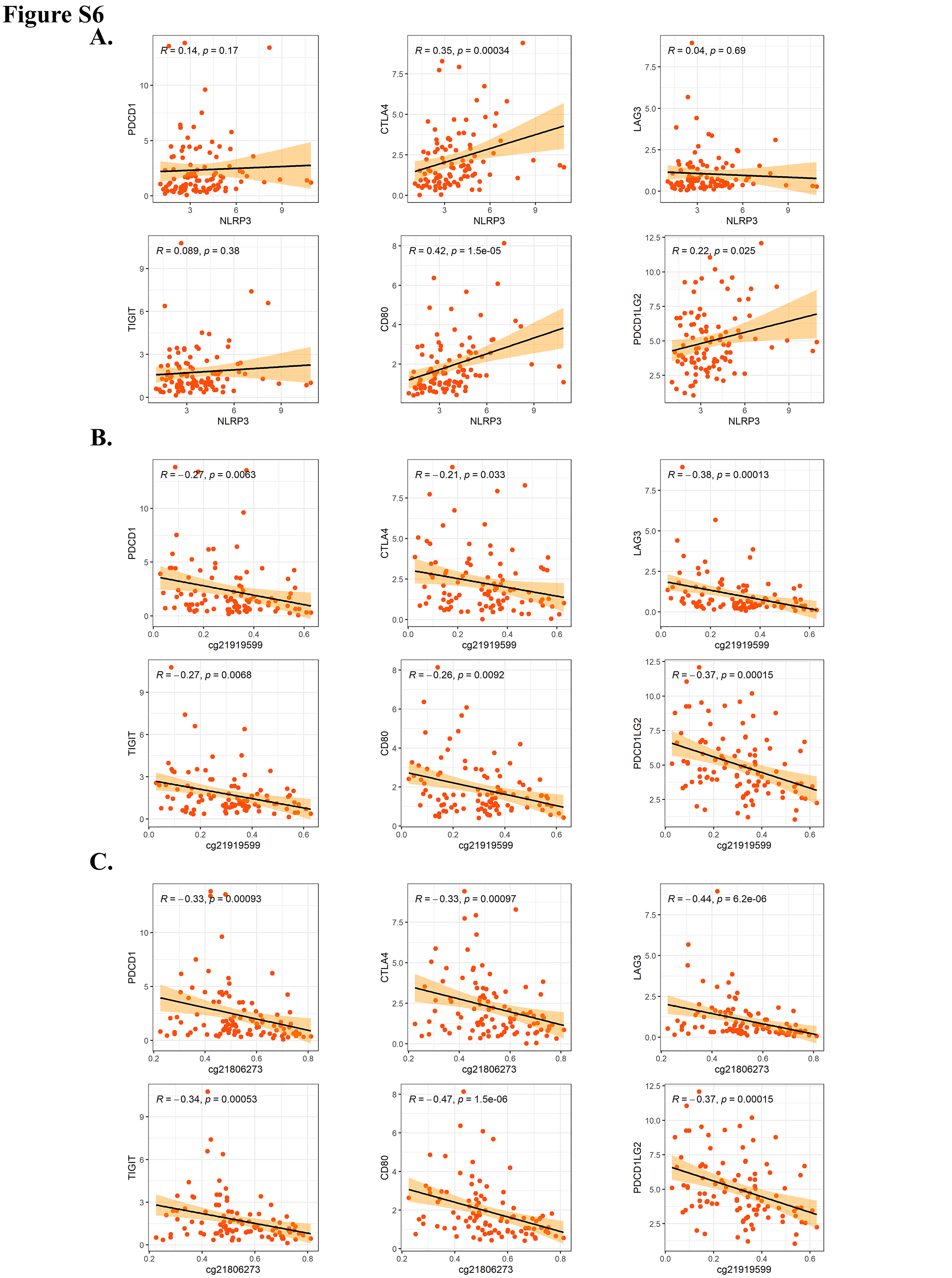

Supplement: Supplementary file 7 — FigureS6 [file CTM2-11-e528-s006.tif]

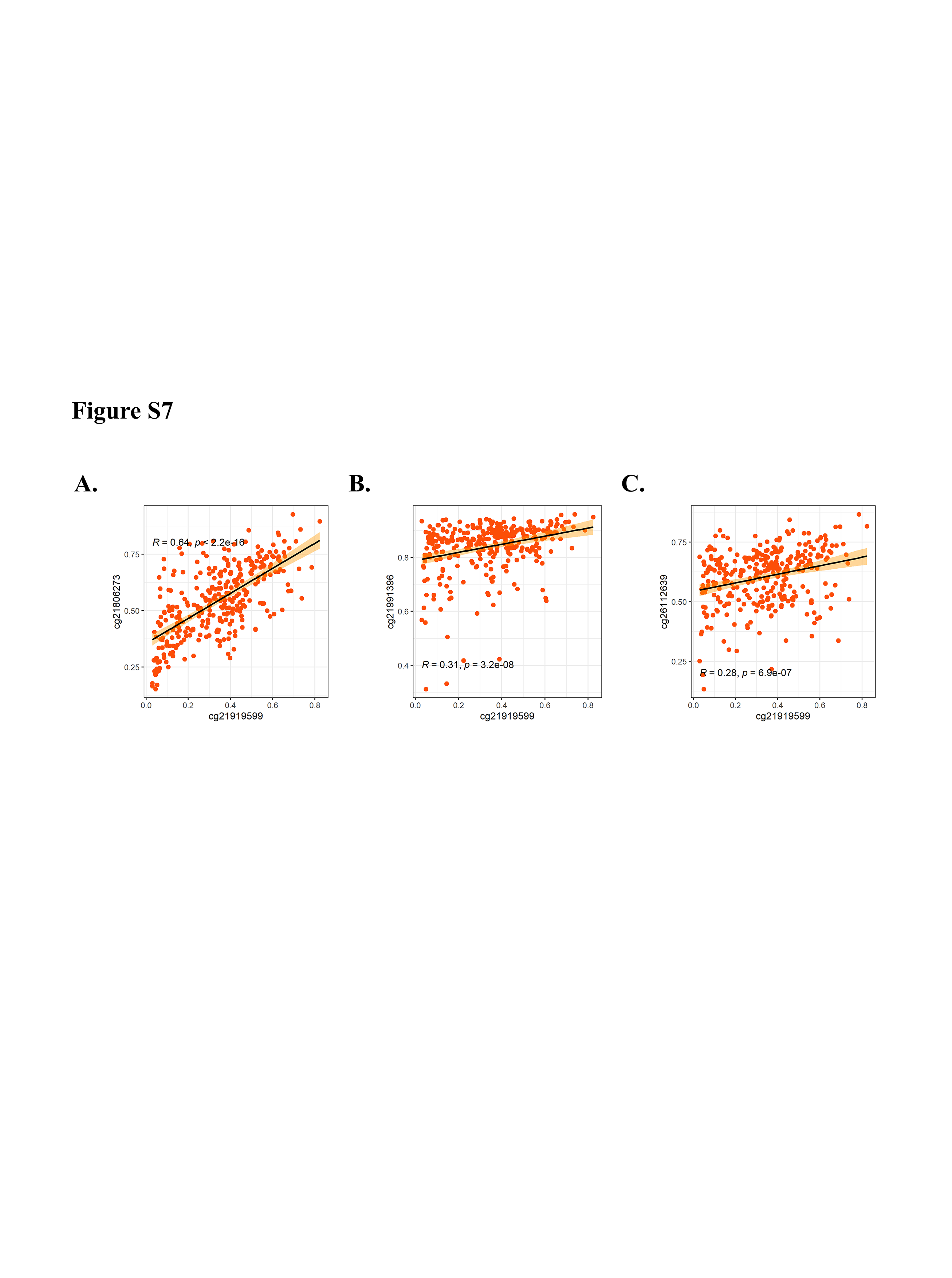

Supplement: Supplementary file 8 — FigureS7 [file CTM2-11-e528-s002.tif]
