# Supplementary material for: Prognostic, clinicopathological, and immune correlation of NLRP3 promoter methylation in kidney renal clear cell carcinoma
Source: Clin Transl Med. 2021 Oct 21;11(10):e528. doi: 10.1002/ctm2.528 (PMC8530444; doi:10.1002/ctm2.528)
Supplement: Supplementary file 9 — Supporting Information [file CTM2-11-e528-s001.docx]

**Material and methods**

**Material and methods**

**Databases and resources**

**TCGA Database**

We downloaded the KIRC related clinical data, gene expression and methylation data from the Cancer Genome Atlas Data Portal (TCGA, http://cancergenome.nih.gov/). The RNA-seq data expressed as transcripts per million (TPM) from TCGA were normalized using R package (edgeR) of R version 4.0.3 software. The relevant methylation data were analyzed by the R package ‘ChAMP’. In terms of differential methylation site analysis, data including 323 tumors and 160 adjacent normal tissues were obtained from Infinium humanmethylization450 BeadChips. Combined methylation and transcriptome analysis were performed for 317 tumor tissues with both methylation and expression data. Clinical and pathological information of TCGA patients were supplemented Table S2.

**CPTAC and GEO datasets**

We downloaded the gene expression data and methylation data from the Clinical Proteomic Tumor Analysis Consortium (CPTAC) of the National Cancer Institute, which included 100 KIRC samples. The expression and methylation data were analyzed in the same way used for TCGA database. The methylation data of GSE70303 and GSE105260 were obtained from the gene expression comprehensive (GEO) database to verify the differential methylation of NLRP3 promoters.

**SYSUCC cohort**

In order to verify the methylation of cg21919599 in KIRC, 109 samples were collected from Sun Yat-sen University Cancer Center (SYSUCC), including 14 normal adjacent tissues and 95 tumor tissues. Patients underwent simultaneous surgery from 2009 to 2015, and clinical follow-up information was collected for analysis. Basic information of datasets included in our study was supplemented in Table S3.

**Methylation analysis**

To get more information of methylation, we downloaded the related methylation data from TCGA and CPTAC databases. Beta value was used to evaluate the methylation levels, and the CpG sites in NLRP3 were analyzed by Infinium HumanMethylation450 BeadChip beads. In order to analyze the differential methylation sites, we chose R package ‘limma’ to normalize the standard of adjusted *P* value < 0.05 and log (fold change) > 0.05. For pan-cancer analysis of NLRP3 promoter methylation, organized data from TCGA was downloaded using “The SMART App”^1^.

In order to verify the methylation of cg21919599 in SYSUCC cohort, we used pyrorelease acid sequencing to check the percentage of methylation. In short, the TIANamp Genomic DNA Kit (# DP304) was applied to extract DNA. After sulfite conversion, specific primers were used to amplify the products containing cg21919599 CpG site by PCR.

Finally, the PCR products were converted to pyrorelease acid sequencing according to the instructions. The PCR primers: forward: 5'- GTTGTAGAGGGTAGTGTTAGAGGTAA -3'; reverse: 5'- AACAAAAAAAATATAATCTCCACTTACAT -3'.

**Antibodies and Immunohistochemistry (IHC)**

All the antibodies used in this study were purchased from servicebio: anti-NLRP3 (#Gb11300), anti-CD4 (#Gb11064), anti-CD8 (#Gb13429). Immunohistochemistry (IHC) was carried out in paraffin tissue sections. In brief, we removed the paraffin and then performed an antigen retrieval procedure. Next, we incubated the fragments with the antibody of NLPR3, CD4 or CD8 in a 4°C humidified container. Then an appropriate amount of secondary antibody was added and incubate at room temperature for 50 minutes. All slides were stained with hematoxylin, dehydrated, installed, and then treated with peroxidase-conjugated vitamin/biotin. Finally, 3DHISTECH (Hungary) was used to scan and digitize all the IHC slides. Quantitative analysis of ion staining was performed using Image Analysis Quantum Center 2.1. According to literature^2^, the ion staining data of NLRP3, CD4 AND CD8 are expressed as density fractions (the total number of positive cells per 1mm^2^ area).

**The relationship between NLRP3 expression/promoter methylation and other biological processes**

Other researchers have constructed a panel of gene sets to store genes related to some biological processes related to tumor immune microenvironment , which includes a variety of genes such as immune checkpoint, CD8 T effector ,Antigen processing machinery, angiogenesis, epithelial mesenchymal transition (EMT) markers, IFN reaction, pan fibroblast TGF β reaction characteristics and co-inhibited APC, and so on^3-6^ (Table S4). Relative of pathway enrichment scores of each sample of TCGA and CPTAC cohort were supplemented in Table S5 and S6, respectively. Then, we explored the enrichment fraction of each biological process related to NLRP3 expression and promoter methylation in high and low groups respectively.

**Genomic set variation analysis (GSVA) and evaluation of immune cell infiltration in tumor microenvironment**

We used the R package "GSVE" to analyze the invasion and biological pathway of immune cells. The GSVE method is unsupervised and nonparametric, and it bypasses the traditional method of explicitly modeling phenotype in affluence scoring algorithm^7^. Other gene sets of other procedures were gained from the Powles, T^3^.

We used the single sample gene set enrichment analysis (ssGSEA) algorithm to quantify the relative level of immune infiltration cells in tumor microenvironment. The gene set of each type of tumor microenvironment infiltrating immune cells came from the study by Zhou, which stores 23 human immune cell subtypes, including activated CD4 and CD8 T cells, activated B cells, etc (Table S7). We used the enrichment fraction reckoned by ssGSEA as the relative abundance of infiltrating immune cells in tumor microenvironment in each sample. Relative abundance of tumor microenvironment cells in each sample of TCGA and CPTAC cohort was supplemented in Table S8 and S9, respectively.

**Statistical analysis**

SPSS (version23.0), GraphPad prism (version 8) and R (version 4.0.3) were used for statistical analysis. In our study we calculated the correlation coefficient by Spearman's rank correlation (Spearman's R). We also used *t* test or Mann Whitney test to compare the differences between groups. In order to carry out survival analysis, the optimal critical value was calculated by R software package ‘survminer’. We also used Kaplan Meier survival analysis to analyze the connection between NLRP3 expression and promoter methylation with overall survival in KIRC. Log rank test was used to determine the significance of the difference. All statistical P values were positive or negative, with *P* < 0.05 as statistically significant* *P* <0.05, ** *P* <0.01, *** *P* <0.001, **** *P* <0.0001.

1. Li Y, Ge D, Lu C. The SMART App: an interactive web application for comprehensive DNA methylation analysis and visualization. *Epigenetics Chromatin*. Dec 5 2019;12(1):71. doi:10.1186/s13072-019-0316-3

2. Sharma A, Subudhi SK, Blando J, et al. Anti-CTLA-4 Immunotherapy Does Not Deplete FOXP3(+) Regulatory T Cells (Tregs) in Human Cancers. *Clin Cancer Res*. Feb 15 2019;25(4):1233-1238. doi:10.1158/1078-0432.CCR-18-0762

3. Mariathasan S, Turley SJ, Nickles D, et al. TGFbeta attenuates tumour response to PD-L1 blockade by contributing to exclusion of T cells. *Nature*. Feb 22 2018;554(7693):544-548. doi:10.1038/nature25501

4. Zhang B, Wu Q, Li B, Wang D, Wang L, Zhou YL. m(6)A regulator-mediated methylation modification patterns and tumor microenvironment infiltration characterization in gastric cancer. *Mol Cancer*. Mar 12 2020;19(1):53. doi:10.1186/s12943-020-01170-0

5. Senbabaoglu Y, Gejman RS, Winer AG, et al. Tumor immune microenvironment characterization in clear cell renal cell carcinoma identifies prognostic and immunotherapeutically relevant messenger RNA signatures. *Genome Biol*. Nov 17 2016;17(1):231. doi:10.1186/s13059-016-1092-z

6. Rosenberg JE, Hoffman-Censits J, Powles T, et al. Atezolizumab in patients with locally advanced and metastatic urothelial carcinoma who have progressed following treatment with platinum-based chemotherapy: a single-arm, multicentre, phase 2 trial. *Lancet*. May 7 2016;387(10031):1909-20. doi:10.1016/S0140-6736(16)00561-4

7. Hanzelmann S, Castelo R, Guinney J. GSVA: gene set variation analysis for microarray and RNA-seq data. *BMC Bioinformatics*. Jan 16 2013;14:7. doi:10.1186/1471-2105-14-7
